# Association of Life’s Crucial 9 with all-cause and cardiovascular mortality among the individuals with chronic inflammatory airway diseases: A prospective cohort study

**DOI:** 10.1097/MD.0000000000050633

**Published:** 2026-09-11

**Authors:** Youli Wen, Wenqiang Li, Jingshan Bai, Guojie Teng, Yanlei Ge, Yuting Fan, Qian Huang

**Affiliations:** a Department of Pulmonary and Critical Care Medicine, Zigong First People’s Hospital, Zigong, Sichuan, China; b Department of Respiratory Medicine, Xiongan Xuanwu Hospital, Xiongan New Area, Baoding, Hebei, China; c Department of Respiratory Medicine, Xuanwu Hospital Capital Medical University, Beijing, China; d Department of Respiratory Medicine, North China University of Science and Technology Affiliated Hospital, Tangshan, Hebei, China; e Department of Clinical Medicine, North Sichuan Medical College, Nanchong, Sichuan, China; f Department of Respiratory Medicine, Dazhou Dachuan District People’s Hospital (Dazhou Third People’s Hospital), Dazhou, Sichuan, China.

**Keywords:** CIAD, Life’s Crucial 9, mortality risk, NHANES

## Abstract

Patients with chronic inflammatory airway diseases (CIAD) have been found to carry a higher burden of cardiovascular diseases. The objective of our research is to explore the link between Life’s Crucial 9 (LC9), a newly introduced measure of cardiovascular health by the American Heart Association, and the risks of all-cause and cardiovascular mortality among patients with CIAD. This prospective cohort study included 2403 patients with CIAD who participated in the National Health and Nutrition Examination Survey from 2007 to 2018. Cox proportional hazards regression models were utilized to calculate the potential associations between LC9 and all-cause mortality and cardiovascular disease mortality in CIAD patients. Restricted cubic spline was employed to estimate possible nonlinear associations. A total of 2403 patients with CIAD were included in the study. The LC9 were divided into 4 quartiles, using the lowest quartile as the reference category for comparison. Following the adjustment for various confounding factors, it was observed that higher LC9 were linked to a reduced risk of all-cause and cardiovascular mortality among the patient population. After adjusting for age, sex, ethnicity, education level, marital status, poverty-to-income ratio, and insurance status, the risk of all-cause mortality decreased by 71% (hazard ratio = 0.29, 95% confidence interval: 0.18–0.49), and the risk of cardiovascular mortality decreased by 87% (hazard ratio = 0.13, 95% confidence interval: 0.05–0.35). Furthermore, the analysis utilizing restricted cubic splines uncovered a nonlinear, positive association between the LC9 scores and mortality rates, including all-cause and cardiovascular mortality, among patients with CIAD. This research indicates that elevated LC9 scores are linked to a lower likelihood of all-cause and cardiovascular mortality in individuals suffering from CIAD.

## 1. Introduction

Chronic inflammatory airway diseases (CIAD), which encompass asthma, chronic bronchitis, and chronic obstructive pulmonary disease (COPD), have become a major public concern.^[1,2]^ Patients affected by these conditions often exhibit characteristic symptoms such as coughing, sputum production, dyspnea, and wheezing, which severely impact their quality of life.^[3]^ Smoking, indoor air pollution from solid fuels, and environmental particulate matter are highlighted as the primary risk factors for chronic respiratory disease-related disabilities in the 2017 Global Burden of Disease report.^[2]^ Industrialization, an aging population, and urbanization are contributing factors that have resulted in a steady rise in both the incidence and mortality associated with a range of chronic respiratory diseases.^[4]^ After cardiovascular diseases (CVDs) and cancer, chronic respiratory ailments became the third highest cause of mortality in 2017, representing 7.0% of total deaths. Among these, asthma and COPD were identified as the leading chronic respiratory conditions.^[5]^ Asthma and COPD are the most common types of chronic respiratory diseases.^[6–8]^ The high prevalence and incidence of these diseases place a substantial economic burden on healthcare systems.^[9,10]^

The pathogenesis of CIAD is complex and influenced by various environmental and genetic factors.^[11,12]^ Research has consistently indicated that individuals afflicted with COPD face a heightened risk of developing CVDs when compared to their counterparts without COPD. Moreover, it has been observed that among COPD patients, the rate of mortality attributed to heart diseases surpasses that of respiratory diseases. Individuals with COPD are estimated to be 2 to 4 times more susceptible to fatal outcomes from CVDs compared to those who do not have COPD.^[13]^ In a randomized controlled trial by Anthonisen NR, it was discovered that out of 5887 asymptomatic smokers with mild to moderate airway obstruction, 731 passed away over a follow-up period of 14.5 years. Within the reported fatalities, a mere 7.8% were attributed to noncancerous respiratory conditions, whereas 22% of the deaths were due to CVDs.^[14]^ These findings underscore the significance of reducing mortality rates among patients with CIAD. They also stress the necessity for a holistic approach to prevent CIAD and enhance initiatives aimed at diminishing the mortality risks associated with this condition.

In 2010, the American Heart Association (AHA) developed a comprehensive index, Life’s Simple 7, based on 7 health behaviors and factors to represent cardiovascular health (CVH).^[15]^ Life’s Simple 7 was created to encourage a transition from treating diseases to actively supporting and sustaining health for both individuals and populations over their lifetimes.^[15]^ In 2022, the AHA revised its definition, incorporating empirical data and practical insights, to include sleep as part of Life’s Essential 8 (LE8). This comprehensive framework encompasses 8 critical health components: the quality of diet, levels of physical exercise, exposure to tobacco, sleep quality, body mass index (BMI), lipid profiles in the blood, blood sugar levels, and blood pressure.^[16]^ However, LE8 lacks an assessment of mental health. Among these, depression is a significant global mental health issue, with an estimated 300 million people affected worldwide.^[17]^ Recent perspectives further underscore the importance of mental health in preventing CVD and introduce a new indicator called Life’s Crucial 9 (LC9), which integrates mental health into LE8.^[18]^

Research is limited regarding the association between LC9 scores and the risk of death in CIAD patients. Therefore, this study aims to investigate the relationship between LC9 scores and all-cause and cardiovascular mortality in CIAD patients. The primary goal of this study is to improve the survival rate of CIAD patients.

## 2. Methods

### 2.1. Study data and study population

Conducted by the National Center for Health Statistics at the United States (US) Centers for Disease Control and Prevention, the National Health and Nutrition Examination Survey (NHANES) is a nationally representative, cross-sectional survey of the civilian, noninstitutionalized US population.^[19]^ This study adhered to the Strengthening the Reporting of Observational Studies in Epidemiology reporting guidelines and involved a secondary analysis of de-identified, publicly available data from NHANES. Consequently, it was exempt from additional institutional review board approval and informed consent requirements.^[20]^

**Figure 1.**
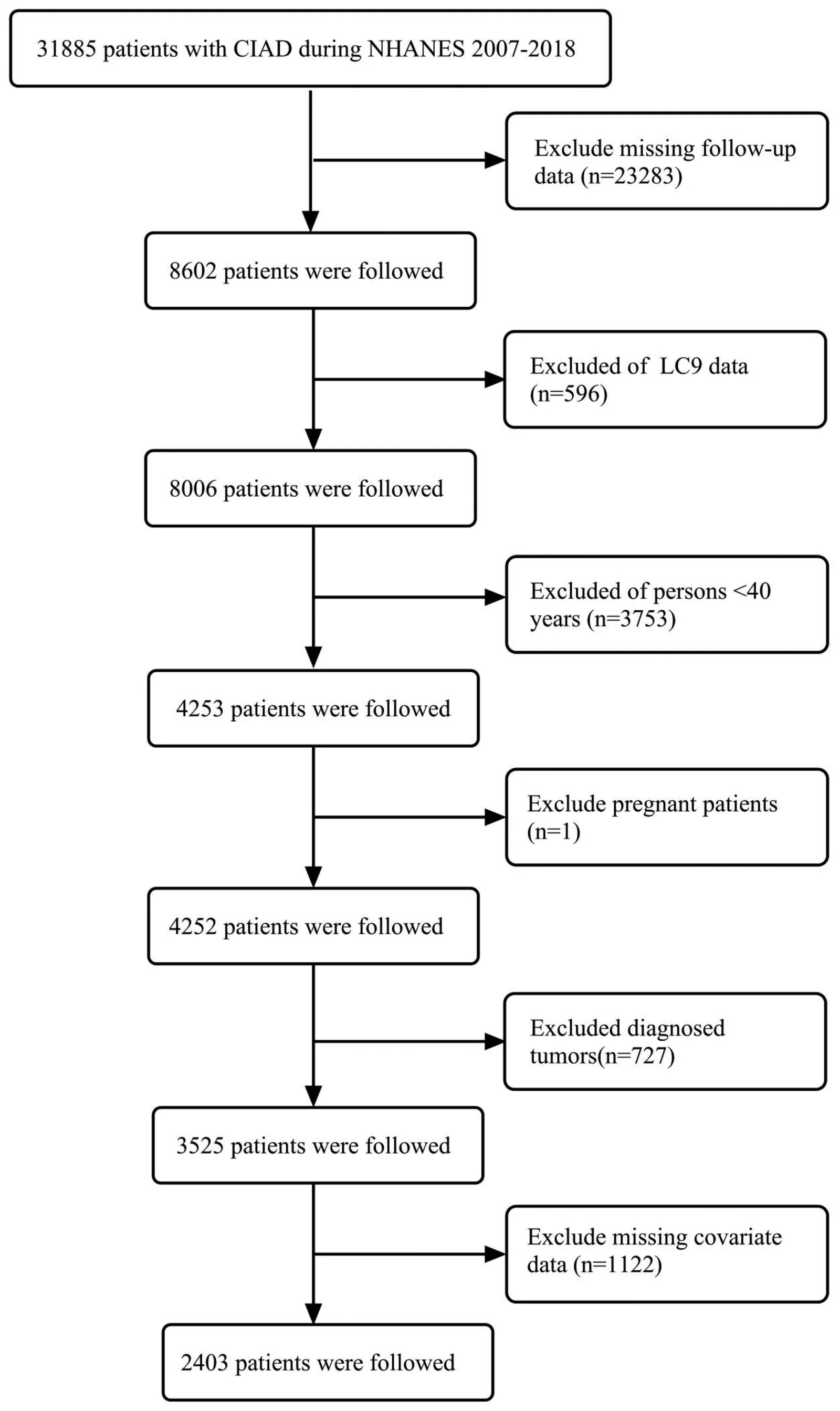
Flowchart of participant selection. CIAD = chronic inflammatory airway diseases, LC9 = Life’s Crucial 9, n = number of patients, NHANES = National Health and Nutrition Examination Survey. CIAD = chronic inflammatory airway diseases, COPD = chronic obstructive pulmonary disease, CVD = cardiovascular disease, CVH = cardiovascular health, HDLC = high-density lipoprotein cholesterol, HR = hazard ratio, LC9 = Life’s Crucial 9, LE8 = Life’s Essential 8, NHANES = National Health and Nutrition Examination Survey, PHQ-9 = Patient Health Questionnaire-9, PIR = poverty-to-income ratio, RCS = restricted cubic spline, US = United States.

Within the scope of this research, we conducted an analysis utilizing data collected from NHANES, spanning the years 2007 to 2018. In this study, we analyzed data from NHANES 2007 to 2018. During these cycles, we included 31,885 CIAD participants, excluded missing follow-up data (n = 23,283), excluded LC9 data (n = 596), excluded persons < 40 years (n = 3,753), excluded pregnant patients (n = 1), excluded diagnosed tumors (n=727), and excluded missing covariate data (n = 1,122). In conclusion, the study comprised a total of 2403 participants (Fig. 1).

### 2.2. Assessment of CIAD

In our research, CIAD encompassed self-reported asthma, chronic bronchitis, and COPD. Participants were queried on the survey whether a physician or healthcare professional had ever informed them that they had asthma, chronic bronchitis, or COPD. Individuals who responded affirmatively to this inquiry were categorized as having CIAD.^[21]^

### 2.3. Measurement of LC9

LC9 is the average of the LE8 score and the depression score, representing a dimension of mental health.^[22]^ The LE8 score encompasses a spectrum of health behaviors and metrics, with 4 behavioral components: diet, physical activity, exposure to nicotine, and sleep duration. Additionally, it includes 4 health indicators: BMI, non-high-density lipoprotein cholesterol (HDLC), blood glucose levels, and blood pressure. The healthy eating index 2015 was utilized to evaluate dietary patterns, based on the 24-hour dietary recall data provided by the participants.^[23]^ Data regarding physical activity, nicotine consumption, sleep habits, history of diabetes, and medication usage were gathered via questionnaires completed by the individuals themselves. Physical examination procedures were used to measure height, weight, and blood pressure, with BMI calculated as weight (in kilograms) divided by height (in meters) squared. Blood samples were taken to evaluate non-HDLC, blood sugar levels, and hemoglobin A1c. Following the sophisticated methodology established by the AHA for assessing CVH, the LE8 score was meticulously derived.^[16]^ Each of the 8 CVH metrics is assigned a score ranging from 0 to 100, and the LE8 total score is calculated as the unweighted average of these 8 metrics.^[24]^

The depression score was calculated based on the Patient Health Questionnaire-9 (PHQ-9) score, which is a validated structured questionnaire for depression screening. Higher PHQ-9 scores indicate higher levels of currently present depressive symptoms. The depression score was assigned as 100, 75, 50, 25, and 0 corresponding to 0 to 4, 5 to 9, 10 to 14, 15 to 19, and 20 to 27 in the PHQ-9 score, respectively.^[25]^ The LC9 score was calculated as the mean of the 8 metrics in the LE8 score and the depression score.^[22]^ LC9 by quartiles (Q1: ≤ 25th percentile (17.78–52.78), Q2: > 25th to 50th percentile (> 52.78–63.33), Q3: > 50th to 75th percentile (> 63.33–72.78), Q4: > 75th percentile (> 78.78–100)) were used as categorical variables.

### 2.4. Covariates

From the questionnaire survey, covariates were extracted, including age (40–64, ≥ 65), sex (Male, Female), ethnicity (White, Others), educational level (< High school, High school diploma, > High school), marital status (Married/Living with Partner, Widowed/Divorced/Separated, Never married), insurance (Yes/No), and poverty-to-income ratio (PIR) (< 1.3, 1.3–3.5, > 3.5). These were used as categorical variables. The PIR is calculated as the ratio of family income to a designated poverty threshold for the survey year, with the threshold established based on the poverty guidelines issued by the Department of health and human services.^[26]^

### 2.5. Statistical analysis

Descriptive statistics depicted categorical variables using total counts, proportions, and percentages. To compare categorical variables between groups, the chi-square test was employed. In accordance with the guidelines for NHANES data analysis, the correct sampling weights were chosen to ensure the accuracy of the analysis (https://wwwn.cdc.gov/nchs/nhanes/tutorials/default.aspx). The associations between LC9 and all-cause and cardiovascular mortality in CIAD were evaluated using weighted Cox regression analysis. Model 1 was unadjusted; Model 2 was adjusted for age, sex, ethnicity, education level, and marital status; and Model 3 added adjustments for PIR and insurance on top of Model 2. Subgroup analyses were conducted to examine the associations and potential interactions between LC9 and all-cause and cardiovascular mortality in CIAD. Furthermore, a restricted cubic spline (RCS) model was implemented to explore and illustrate the potential dose-response associations between the LC9 score and mortality outcomes, encompassing both all-cause and cancer-specific mortality, within the cohort of CIAD patients. The analysis was conducted using R statistical software (version 4.3.3; R Foundation for Statistical Computing, Vienna, Austria; https://cran.r-project.org/). In all analyses, a 2-tailed *P* value of < .05 was deemed to indicate statistical significance.

## 3. Results

### 3.1. Baseline characteristics of LC9 and all-cause mortality in CIAD

Table 1 presents the baseline characteristics of the cohort study participants (n = 2403) grouped by the survival status of CIAD patients, with 338 patients dying from all-cause mortality. Among the 2403 CIAD patients aged 40 years and older, the all-cause mortality group was more likely to include patients who were ≥ 65 years old, White, Married/Living with Partner, with education levels > High school, PIR 1.3 to 3.5, and in the LC9 Q1 group. There was no significant difference in all-cause mortality between males and females (Table 1).

**Table 1:** Baseline characteristics of LC9 and all-cause mortality in CIAD patients.

Variable	Total	CIAD patients		*P* value
		Survival	Death	
Age, n (%)				< .0001
40–64 yrs	1644 (74.55)	1519 (78.52)	125 (39.62)	
≥ 65 yrs	759 (25.45)	546 (21.48)	213 (60.38)	
Sex, n (%)				.02
Female	1356 (57.81)	1203 (58.68)	153 (50.20)	
Male	1047 (42.19)	862 (41.32)	185 (49.80)	
Ethnicity, n (%)				.004
White	1242 (75.12)	1020 (74.29)	222 (82.45)	
Others	1161 (24.88)	1045 (25.71)	116 (17.55)	
Marital status, n (%)				< .001
Never married	211 (7.88)	184 (7.87)	27 (7.94)	
Married/Living with Partner	1383 (65.16)	1226 (66.72)	157 (51.48)	
Widowed/Divorced/Separated	809 (26.96)	655 (25.41)	154 (40.58)	
Educational level, n (%)				< .0001
< High school	606 (16.49)	496 (15.31)	110 (26.83)	
High school diploma	571 (24.50)	479 (24.16)	92 (27.50)	
> High school	1226 (59.01)	1090 (60.52)	136 (45.67)	
PIR, n (%)				< .0001
< 1.3	850 (24.15)	702 (22.74)	148 (36.53)	
1.3–3.5	888 (33.46)	746 (32.41)	142 (42.68)	
> 3.5	665 (42.39)	617 (44.84)	48 (20.80)	
Insurance, n (%)				.09
No	306 (11.51)	277 (11.88)	29 (8.26)	
Yes	2097 (88.49)	1788 (88.12)	309 (91.74)	
LC9, n (%)				< .0001
Q1 (17.78–52.78)	622 (20.80)	495 (19.08)	127 (35.92)	
Q2 (52.78–63.33)	579 (21.83)	490 (21.56)	89 (24.20)	
Q3 (63.33–72.78)	608 (26.24)	530 (26.27)	78 (26.00)	
Q4 (78.78–100_)	594 (31.13)	550 (33.09)	44 (13.88)	

CIAD = chronic inflammatory airway diseases, LC9 = Life’s Crucial 9, n = number of patients, PIR = poverty-to-income ratio.

### 3.2. Association of LC9 with all-cause mortality in CIAD

During a median follow-up period of 79 months, 338 out of 2403 CIAD patients died from various causes. In Model 1, compared to the LC9 lowest quartile (Q1) group, the risk of all-cause mortality in CIAD patients showed a decreasing trend with increasing quartiles (*P* for trend < .0001), with the highest quartile (Q4) group experiencing a 77% reduction in the risk of all-cause mortality (hazard ratio [HR] = 0.23, 95% confidence interval [CI]: 0.13–0.40). After adjusting for multiple factors, the association between high LC9 levels and reduced risk of all-cause mortality in CIAD patients persisted in Models 2 and 3, with risks reduced by 76% and 71%, respectively (Model 2, HR = 0.24, 95% CI: 0.14–0.41; Model 3, HR = 0.29, 95% CI: 0.18–0.49) (Table 2).

**Table 2:** Cox regression of the association between LC9 and all-cause mortality in CIAD patients.

	Model 1	Model 2	Model 3
	HR (95% CI)	HR (95% CI)	HR (95% CI)
LC9			
Q1	Ref.	Ref.	Ref.
Q2	0.67 (0.45–1.00)	0.59 (0.40–0.87)	0.62 (0.42–0.91)
Q3	0.53 (0.33–0.86)	0.48 (0.33–0.72)	0.54 (0.38–0.78)
Q4	0.23 (0.13–0.40)	0.24 (0.14–0.41)	0.29 (0.18–0.49)
*P* for trend	< .0001	< .0001	< .0001

Data were present as HR and 95% CI.

Model 1: unadjusted models.

Model 2: adjusted for age, sex, ethnicity, education level, and marital status.

Model 3: adjusted for age, sex, ethnicity, education level, marital status, PIR, and insurance.

CI = confidence interval, CIAD = chronic inflammatory airway diseases, HR = hazard ratio, LC9 = Life’s Crucial 9, PIR = poverty-to-income ratio, Ref = reference.

**Figure 2.**
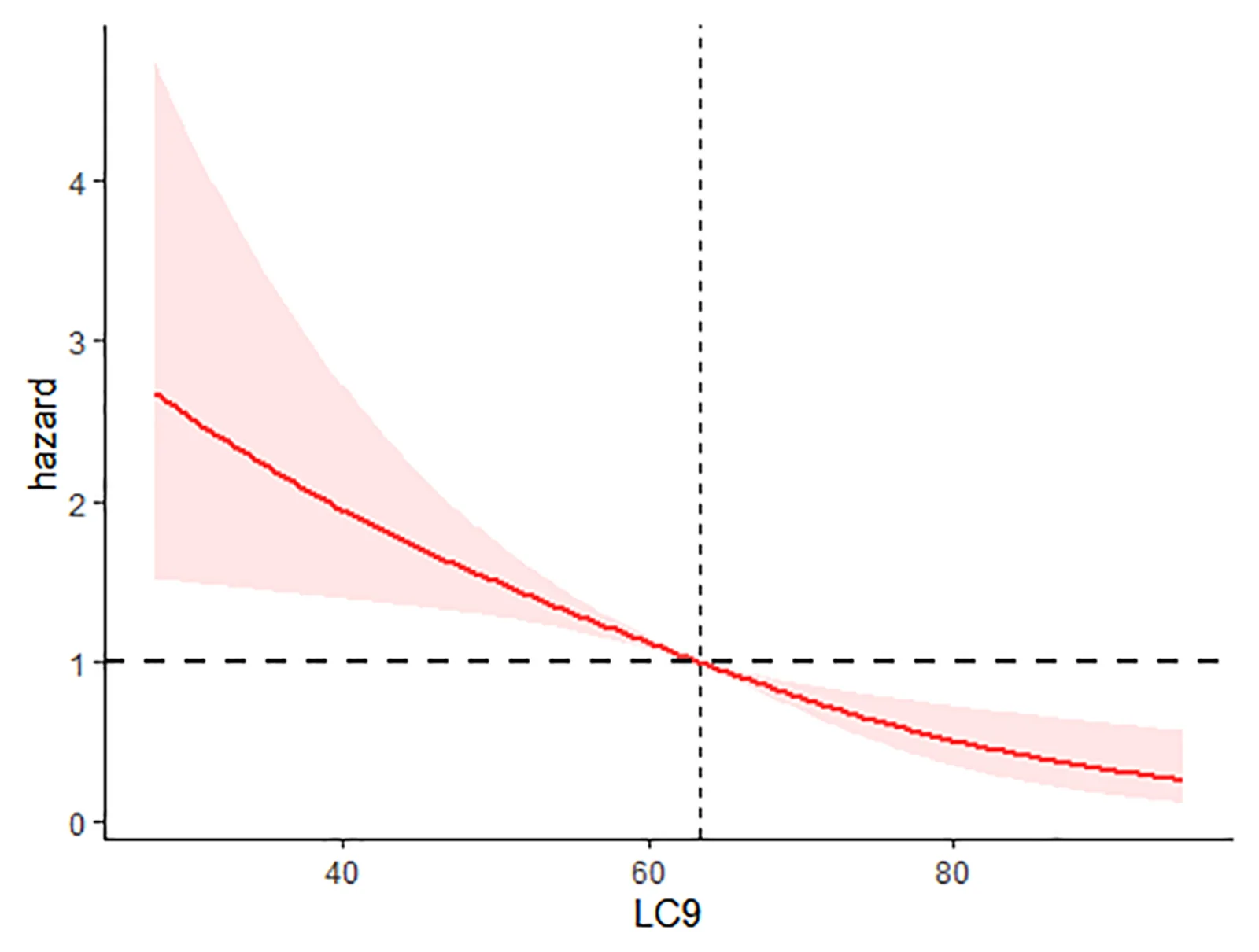
Correlation between LC9 and all-cause mortality risk in CIAD patients. CIAD = chronic inflammatory airway diseases, LC9 = Life’s Crucial 9.

Analysis using RCS curves indicated a decreasing trend between LC9 and all-cause mortality in CIAD patients (Fig. 2).

Subgroup analyses based on age, sex, race, and PIR were conducted. The results showed that the decreasing trend between LC9 and cardiovascular mortality risk in CIAD patients was consistent with the findings described above. No significant interaction between subgroups was observed (*P* > .05) (Table 3).

**Table 3:** Stratified analysis of the association between LC9 and all-cause mortality in CIAD patients.

Character	Q1	Q2	*P*	Q3	*P*	Q4	*P*	*P* for trend	*P* for interaction
Sex									.38
Female	Ref	0.73 (0.38–1.40)	.34	0.73 (0.36–1.47)	.38	0.23 (0.10–0.53)	< .001	< .001	
Male	Ref	0.60 (0.39–0.92)	.02	0.36 (0.21–0.63)	< .001	0.21 (0.11–0.41)	< .0001	< .0001	
Age									.3
40–64 yrs	Ref	0.44 (0.25–0.77)	.004	0.35 (0.18–0.68)	.002	0.14 (0.05–0.39)	< .001	< .0001	
≥ 65 yrs	Ref	0.74 (0.45–1.21)	.22	0.62 (0.38–1.02)	.06	0.33 (0.18–0.59)	< .001	< .0001	
Ethnicity									.8
White	ref	0.68 (0.41–1.12)	.13	0.53 (0.30–0.95)	.03	0.23 (0.12–0.44)	< .0001	< .0001	
Others	ref	0.54 (0.29–1.01)	.05	0.48 (0.27–0.88)	.02	0.14 (0.06–0.33)	< .0001	< .0001	
PIR									.42
< 1.3	ref	0.80 (0.42–1.55)	.51	0.82 (0.42–1.62)	.57	0.30 (0.13–0.67)	.003	.01	
1.3–3.5	ref	0.92 (0.54–1.57)	.76	0.57 (0.32–1.01)	.05	0.27 (0.14–0.52)	< .0001	< .001	
> 3.5	ref	0.36 (0.15–0.85)	.02	0.36 (0.15–0.87)	.02	0.28 (0.11–0.74)	.01	.04	

CIAD = chronic inflammatory airway diseases, LC9 = Life’s Crucial 9, PIR = poverty-to-income ratio, ref = reference.

### 3.3. Baseline characteristics of LC9 and cardiovascular mortality in CIAD

Table 4 displays the initial characteristics of the cohort study participants (n = 2403) divided into LC9 quartiles, with 127 patients having died from cardiovascular causes. Among the 2403 CIAD patients aged 40 years and older, cardiovascular mortality in CIAD patients was more likely to occur in individuals who were ≥ 65 years old, White, Married/Living with Partner, had education levels > High school, PIR < 1.3, and were in the LC9 Q1 group (Table 4).

**Table 4:** Baseline characteristics of LC9 and cardiovascular mortality in CIAD patients.

Variable	Total	CIAD patients		*P* value
		Survival	Death	
Age, n (%)				< .0001
40–64 yrs	1565 (77.05)	1519 (78.52)	46 (39.81)	
≥ 65 yrs	627 (22.95)	546 (21.48)	81 (60.19)	
Sex, n (%)				.06
Female	1259 (58.29)	1203 (58.68)	56 (48.44)	
Male	933 (41.71)	862 (41.32)	71 (51.56)	
Ethnicity, n (%)				.04
White	1103 (74.59)	1020 (74.29)	83 (82.22)	
Others	1089 (25.41)	1045 (25.71)	44 (17.78)	
Marital status, n (%)				< .001
Never married	197 (7.83)	184 (7.87)	13 (6.69)	
Married/Living with Partner	1279 (66.06)	1226 (66.72)	53 (49.43)	
Widowed/Divorced/Separated	716 (26.11)	655 (25.41)	61 (43.88)	
Educational level, n (%)				.002
< High school	541 (15.84)	496 (15.31)	45 (29.31)	
High school diploma	510 (24.10)	479 (24.16)	31 (22.60)	
> High school	1141 (60.05)	1090 (60.52)	51 (48.09)	
PIR, n (%)				.01
< 1.3	760 (23.37)	702 (22.74)	58 (39.24)	
1.3–3.5	792 (32.45)	746 (32.41)	46 (33.37)	
> 3.5	640 (44.18)	617 (44.84)	23 (27.39)	
Insurance, n (%)				.85
No	291 (11.85)	277 (11.88)	14 (11.22)	
Yes	1901 (88.15)	1788 (88.12)	113 (88.78)	
LC9, n (%)				< .0001
Q1 (17.78–52.78)	551 (19.91)	495 (19.08)	56 (40.92)	
Q2 (52.78–63.33)	520 (21.54)	490 (21.56)	30 (21.00)	
Q3 (63.33–72.78)	560 (26.39)	530 (26.27)	30 (29.62)	
Q4 (78.78–100)	561 (32.15)	550 (33.09)	11 (8.45)	

CIAD = chronic inflammatory airway diseases, LC9 = Life’s Crucial 9, n = number of patients, PIR = poverty-to-income ratio.

### 3.4. Association of LC9 with cardiovascular mortality in CIAD

During a median follow-up period of 79 months, 127 out of 2403 CIAD patients died from cardiovascular causes. In Model 1, compared to the group with the lowest LC9 quartile (Q1), there was a decreasing trend in the risk of cardiovascular mortality as the quartiles increased among CIAD patients (*P* for trend < .0001). The risk of cardiovascular mortality decreased by 89% in the highest quartile (Q4) group (HR = 0.11, 95% CI: 0.04–0.33). After adjusting for multiple factors, the relationship between high LC9 levels and the risk of CIAD cardiovascular mortality persisted in Model 2 and Model 3, with the risk of cardiovascular mortality decreasing by 89% and 87%, respectively (Model 2, HR = 0.11, 95% CI: 0.04–0.31; Model 3, HR = 0.13, 95% CI: 0.05–0.35) (Table 5).

**Table 5:** Cox regression of the association between LC9 and cardiovascular mortality in CIAD patients.

	Model 1	Model 2	Model 3
	HR (95% CI)	HR (95% CI)	HR (95% CI)
Life’s Crucial 9			
Q1	Ref.	Ref.	Ref.
Q2	0.50 (0.27–0.93)	0.43 (0.23–0.78)	0.45 (0.25– 0.83)
Q3	0.51 (0.25–1.01)	0.42 (0.22–0.83)	0.46 (0.24– 0.91)
Q4	0.11 (0.04–0.33)	0.11 (0.04–0.31)	0.13 (0.05– 0.35)
*P* for trend	< .0001	< .0001	< .0001

Data were presented as HR and 95% CI.

Model 1: unadjusted models.

Model 2: adjusted for age, sex, ethnicity, education level, and marital status.

Model 3: adjusted for age, sex, ethnicity, education level, marital status, PIR, and insurance.

CI = confidence interval, CIAD = chronic inflammatory airway diseases, HR = hazard ratio, LC9 = Life’s Crucial 9, PIR = poverty-to-income ratio, Ref = reference.

**Figure 3.**
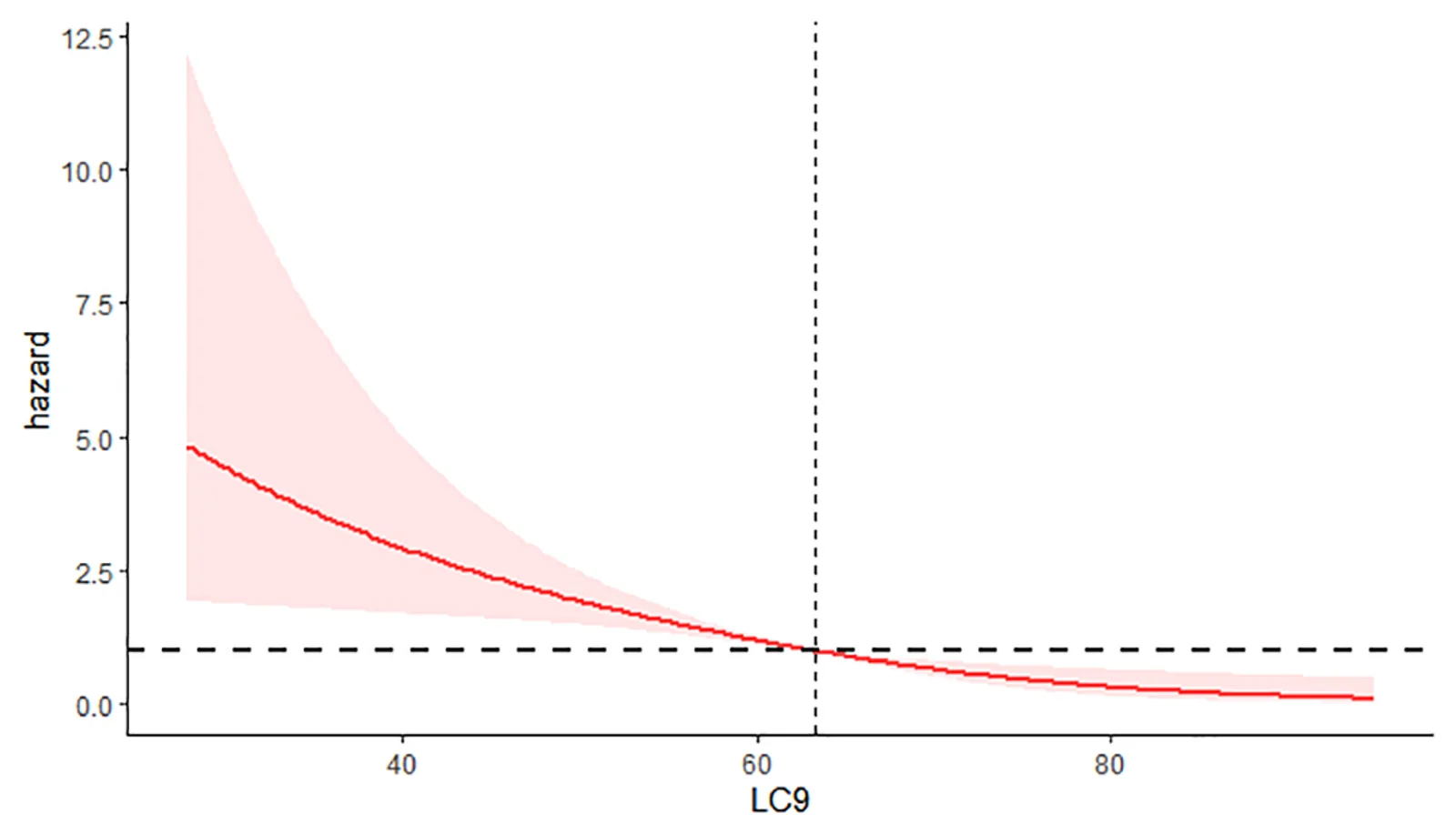
Correlation between LC9 and cardiovascular mortality risk in CIAD patients. CIAD = chronic inflammatory airway diseases, LC9 = Life’s Crucial 9.

Our analysis using RCS demonstrated a decreasing trend between LC9 and cardiovascular mortality in CIAD patients (Fig. 3).

We conducted subgroup analyses based on age, sex, race, and educational level, and the results indicated that the decreasing trend between LC9 and cardiovascular mortality risk in CIAD patients was consistent across these subgroups, with no significant interaction between subgroups (*P* > .05) (Table 6).

**Table 6:** Stratified analysis of the association between LC9 and cardiovascular mortality in CIAD patients.

Character	Q1	Q2	*P*	Q3	*P*	Q4	*P*	*P* for trend	*P* for interaction
Sex									.71
Female	ref	0.57 (0.29–1.11)	.1	0.41 (0.16–1.04)	.06	0.13 (0.04–0.48)	.002	< .001	
Male	ref	0.43 (0.18–1.03)	.06	0.57 (0.24–1.36)	.2	0.08 (0.02–0.30)	< .001	< .001	
Age									.32
40–64 yrs	ref	0.25 (0.09–0.66)	.005	0.25 (0.08–0.73)	.01	0.09 (0.01–0.62)	.02	.005	
≥ 65 yrs	ref	0.65 (0.33–1.31)	.23	0.66 (0.31–1.43)	.29	0.14 (0.05–0.38)	< .0001	< .001	
Ethnicity									.99
White	ref	0.49 (0.24–1.02)	.06	0.50 (0.23–1.12)	.09	0.11 (0.03–0.37)	< .001	< .001	
Others	ref	0.46 (0.17–1.23)	.12	0.43 (0.16–1.18)	.1	0.12 (0.04–0.37)	< .001	.001	
Educational level									.09
< High school	ref	1.47 (0.73–2.92)	.28	0.43 (0.14–1.32)	.14	0.24 (0.08–0.72)	.01	.003	
High school diploma	ref	0.27 (0.10–0.72)	.01	0.50 (0.18–1.36)	.17	0.10 (0.02–0.39)	.001	.002	
> High school	ref	0.23 (0.05–1.11)	.07	0.46 (0.12–1.71)	.24	0.09 (0.01–0.54)	.01	.03	

CIAD = chronic inflammatory airway diseases, LC9 = Life’s Crucial 9, ref = reference.

## 4. Discussion

Drawing from the NHANES cycle data spanning from 2007 to 2018, which encompasses 2403 CIAD patients, this research identified a nonlinear association between LC9 scores and both all-cause and cardiovascular mortality rates among individuals with CIAD. Elevated LC9 scores correlated with a diminished risk of both all-cause and cardiovascular mortality among individuals with CIAD. Stratified analyses indicated that the relationship between LC9 scores and mortality risk in CIAD patients remained consistent across different stratification factors. These findings underscore the importance of higher LC9 scores in improving both overall and CVH outcomes in CIAD patients.

CIAD is a group of common chronic conditions, including COPD, asthma, emphysema, chronic bronchitis, and others.^[27]^ Affected patients often exhibit characteristic symptoms such as coughing, sputum production, shortness of breath, and wheezing, which significantly impair their quality of life.^[28]^ In 2017, approximately 3.91 million individuals succumbed to chronic respiratory diseases, marking an 18% rise compared to 1990 figures. Respiratory ailments of a chronic nature are now ranked as the third most common cause of mortality worldwide, trailing only behind cardiovascular conditions and cancer.^[29]^ COPD and asthma are recognized as the principal categories of chronic respiratory illnesses. In 2019, COPD affected 212.3 million individuals globally, leading to 3.3 million fatalities and accounting for 74.4 million disability-adjusted life years.^[30]^ Furthermore, asthma, a heterogeneous clinical condition, impacts over 300 million individuals worldwide, with 25 million of those cases occurring in the US.^[31]^ These findings reveal that CIAD patients bear a significant disease burden, which urgently necessitates the introduction of effective indicators to predict mortality risk.

LC9, which stands for LC9, is a new health assessment metric that adds a dimension of mental health to the original LE8.^[22]^ The LE8 score encompasses a total of 8 determinants, comprising both lifestyle factors and physiological indicators. Specifically, it accounts for 4 health-related behaviors, which include dietary habits, levels of physical exercise, exposure to nicotine, and the duration of sleep. Additionally, it incorporates 4 physiological metrics, namely BMI, HDLC in nonobese individuals, blood glucose levels, and blood pressure.^[32]^ The LC9 score is the average of the LE8 score and the depression score, with the depression score representing a dimension of mental health. New research consistently indicates that the LE8 score correlates with a multitude of health outcomes. These encompass CVH, the incidence of nonalcoholic fatty liver disease, chronic kidney disease, dementia, and overall life expectancy, as well as the risk of all-cause mortality.^[33–37]^ The LE8 score’s association with these various health metrics highlights its potential as a predictive tool for assessing and mitigating health risks across a spectrum of conditions. Preliminary research has identified substantial correlations, indicating that elevated LE8 scores correlate with extended life expectancies for adults in the US.^[38]^ An independent study has corroborated these results, highlighting a distinct link between elevated LE8 scores and diminished risks of all-cause mortality and cardiovascular mortality among US adults.^[39]^ Multiple studies have evaluated the association between LE8 scores and all-cause and cardiovascular mortality among US adults.^[39–41]^ Higher LE8 scores are independently associated with lower risks of all-cause and cardiovascular mortality. There is an approximately linear dose-response relationship between increases in overall CVH index scores and reductions in both all-cause and cardiovascular mortality.^[40]^ Analysis of these studies indicates that LE8 scores are closely related to all-cause mortality and cardiovascular mortality. Both Zhang et al and Yi et al demonstrated that higher LE8 scores are independently associated with reduced all-cause mortality and cardiovascular mortality.^[40,42]^ Recent research investigated the connection between LE8 and mortality in Spanish adults, finding that increased LE8 scores are related to a reduction in all-cause and cardiovascular mortality.^[43]^ Isiozor et al studied the link between LE8 and mortality risks in Finnish men, finding that optimal CVH (as per LE8) significantly cut risks of coronary heart disease, stroke, and CVD.^[44]^ Multiple studies across various cohorts have validated the effectiveness of the LE8 in forecasting the risks of all-cause and cardiovascular mortality in the broader population.^[40,41,44–46]^ Studies have identified factors associated with LE8 scores, such as poor diet, lack of exercise, smoking, insufficient sleep, and diabetes, which significantly increase the risk of all-cause mortality.^[40]^

Beyond the modifiable domains captured by LC9 (lifestyle, metabolism, and mental health) recent studies pointed to thoracic morphology as an independent correlate of cardiovascular vulnerability. The Modified Haller Index, or the ratio of chest transverse to anteroposterior diameter, identifies individuals with a relatively narrow thoracic cage. Notably, a narrow anteroposterior thoracic diameter (Modified Haller Index > 2.5) is associated with a significantly reduced risk of adverse cardiovascular events.^[47,48]^ These findings raise the possibility that chest shape captures underlying cardiopulmonary mechanics or constitutional traits that affect cardiac function. Such a narrow-chest phenotype may act as an innate protective factor, potentially interacting with or modifying the effects of LC9-related exposures. In CIAD patients, who commonly have airflow obstruction and hyperinflation, the mechanical interplay between chest wall geometry and cardiac performance could be especially relevant. We thus suspect that the mortality benefit associated with higher LC9 scores may in part reflect a synergistic effect; those with both favorable LC9 profiles and a narrow anteroposterior chest phenotype show the lowest cardiovascular risk. Framing LC9 within this broader biological context moves it beyond a purely modifiable score and toward a metric that intersects with constitutive phenotypic variation, offering a more nuanced view of cardiovascular risk in CIAD populations.

Moreover, given that the disease course for CIAD patients is often prolonged, mental health has increasingly attracted public attention. The World Health Organization predicts that by 2030, major depressive disorder will be the leading cause of the global burden of chronic diseases.^[49]^ Some meta-analyses have reported that depression is associated with an increased risk of CVD incidence and mortality, independent of traditional risk factors.^[50–53]^ In a meta-analysis involving 893,850 participants, depression was associated with a 30% increased risk of myocardial infarction and coronary heart disease after adjusting for demographic information and health behaviors.^[50]^ A meta-analysis summarizing the results of 28 prospective cohort studies showed that depression is an independent risk factor for stroke.^[54]^ These 2 meta-analyses included the general population aged 18 years or older, with no specific exclusion criteria. A separate meta-analysis including 198,589 community residents aged 60 years or older showed that late-life depression is independently associated with a significant increase in cardiovascular and all-cause mortality risk.^[53]^ This is consistent with our findings that a higher LC9 score is associated with reduced all-cause and cardiovascular mortality in CIAD patients.

This research offers substantial merits and acknowledges certain constraints, thereby enhancing the depth of our insights. Initially, the inclusion of NHANES data strengthens the external validity of our study outcomes, amplifying their potential for wider application. Subsequently, the development of a novel LC9 score to evaluate the correlation between CIAD and mortality from all causes and CVD enriches our study’s depth and establishes a foundation for deciphering the determinants of mortality among individuals with CIAD. However, it is important to acknowledge some limitations. Firstly, data on diet, physical activity, nicotine exposure, and sleep health in the LC9 score are based on self-reported patient information, which may introduce recall bias. Additionally, despite multivariable adjustments, residual confounding factors may still influence the outcomes. Furthermore, due to sample size limitations, the racial/ethnic group analysis only included White, Black, and Mexican participants, which may limit the generalizability of the results to other groups. Consequently, we clarify that our study’s results are specific to the population under investigation and do not constitute a nationally representative sample. Nevertheless, our research retains a degree of innovation and contributes significantly to addressing pertinent issues in the prevention and management of CIAD patients.

## 5. Conclusion

The study reveals that elevated LC9 scores correlate with a decreased likelihood of all-cause and cardiovascular mortality among individuals suffering from CIAD.

## Acknowledgments

We would like to thank the US Centers for Disease Control and Prevention for collecting the data and making the data available to the public.

## Author contributions

**Conceptualization:** Youli Wen, Wenqiang Li, Jingshan Bai, Guojie Teng, Yuting Fan, Qian Huang.

**Data curation:** Youli Wen, Wenqiang Li, Jingshan Bai, Guojie Teng, Yuting Fan, Qian Huang.

**Formal analysis:** Youli Wen, Wenqiang Li, Jingshan Bai, Guojie Teng, Yuting Fan, Qian Huang.

**Funding acquisition:** Youli Wen, Qian Huang.

**Investigation:** Youli Wen.

**Methodology:** Youli Wen, Wenqiang Li, Jingshan Bai, Yanlei Ge.

**Project administration:** Youli Wen, Qian Huang.

**Resources:** Youli Wen, Qian Huang.

**Software:** Wenqiang Li, Jingshan Bai, Yuting Fan.

**Supervision:** Youli Wen, Yanlei Ge, Qian Huang.

**Validation:** Guojie Teng, Yanlei Ge.

**Visualization:** Wenqiang Li, Jingshan Bai.

**Writing – review & editing:** Youli Wen, Qian Huang.

**Writing – original draft:** Wenqiang Li, Jingshan Bai.
